# Arsenical Poisoning by Paper-Hangings

**Published:** 1860-05

**Authors:** 


*Arsenical Poisoning by Paper-Hangings.*—Three children near Tipton have suffered from the arsenical emanations from a green bed-room paper in a newly-papered house. The symptoms were emaciation, pining, general restlessness, (worse at night,) and twitching of the facial muscles. Dr. Balenden, observing these symptoms, concluded that they were suffering from the effects of gradual poisoning; and, on being removed into another room, the children recovered.—*London Lancet.*

